# MiR-146a Regulates SOD2 Expression in H_2_O_2_ Stimulated PC12 Cells

**DOI:** 10.1371/journal.pone.0069351

**Published:** 2013-07-23

**Authors:** Guohua Ji, Ke Lv, Hailong Chen, Tingmei Wang, Yanli Wang, Dingsheng Zhao, Lina Qu, Yinghui Li

**Affiliations:** State Key Laboratory of Space Medicine Fundamentals and Application, China Astronaut Research and Training Center, Beijing, China; Hertie Institute for Clinical Brain Research and German Center for Neurodegenerative Diseases, Germany

## Abstract

SOD2 (superoxide dismutase 2) is one of the endogenous antioxidant enzymes that protect against reactive oxygen species. While explorations of SOD2 expression regulation are mainly focused on transcriptional and post-translational activation, there are few reports about the post-transcriptional regulation of SOD2. MicroRNAs (miRNAs) are 21nt-25nt (nucleotide) small noncoding RNAs that have emerged as indispensable regulators of gene expression. Here we show that miR-146a, a widely expressed miRNA, is up-regulated by H_2_O_2_-induced stress. By sequence analysis we found a binding site for miR-146a in the *sod2* mRNA 3′UTR, and a luciferase reporter assay confirmed that miR-146a can interact with this *sod2* regulatory region. Our results further show that miR-146a could down-regulate the SOD2 protein expression, and antisense-miR-146a could reverse the decrease of both the SOD2 level and cell viability in H_2_O_2_ treated PC12 cells. In conclusion, here we have identified a novel function of miR-146a in the post-transcriptional regulation of SOD2 expression.

## Introduction

It is well known that the oxidative stress is involved in a lot of diseases like tumors [1], Alzheimer disease [2], aging [3], arthritis [4], neurodegenerative disease [5] and atherosclerosis [6]. The unbalance between oxidation and antioxidation is the mechanism of oxidative stress [7]. *In vivo*, major antioxidases include superoxide dismutase (SOD), glutathione peroxidase (GPx), catalase (CAT) and thioredoxin system [7], [8].

There are three subtypes of SOD in humans, Cu, Zn-SOD (SOD1), Mn-SOD (SOD2), and extra cellular SOD (SOD3) [9]. SOD2 is a mitochondrial manganese (Mn) containing enzyme, which is composed of a 96 kDa homotetramer and localized in the mitochondrial matrix [10]. Mn at the active site of SOD2 serves to catalyze the disproportionation of superoxide anion to oxygen and H_2_O_2_ in a similar fashion as SOD1 and SOD3 [11]. SOD2 was reported to be one of the antioxidases that have anti-tumor effect [12]. Studies have shown that nuclear factor κB (NF-κB) and activator protein (AP-1) can promote *sod2* expression transcriptionally [13]. SIRT3 is a deacetylase located in mitochondria, and a few studies showed that SIRT3 could deacetylate two critical lysine residues on SOD2 and promote its antioxidative activity [14], [15]. But up to now, there have rare reports about the post transcriptional regulation of *sod2* gene expression.

MicroRNAs (miRNAs) are evolutionarily conserved 21nt-25nt (nucleotide) small non-coding RNAs. They bind to partially complementary target sites in messenger RNA (mRNA) 3′-untranslated regions (3′-UTRs), which results in degradation of the target mRNA, or translational repression of the encoded protein [16]. It has been estimated that approximately 60% of all mRNA are under the control of miRNA [17]. MiRNAs play a significant role in various cellular processes including development, differentiation, cell growth, morphogenesis, apoptosis, and neurological disorders [18], [19]. MiR-146a is widely expressed in different species and tissues, and studies have shown that miR-146a was involved in immunity, inflammation and viral infections by regulating different target genes [20]. Lukiw et al., showed that miR-146a was significantly up-regulated in interleukin-1β, Aβ42-, and/or oxidatively-stressed human neural (HN) cells in primary culture, and a consequence of miR-146a up-regulation was the down-regulation of the important immune system regulator complement factor H (CFH) [21]. Another study shows that miR-146a was up-regulated significantly by ROS-generating metal sulfates (iron- plus aluminum-sulfate) in human astroglial (HAG) cells [22].

To reveal the possible involvement of miRNAs in the process of SOD2 expression regulation, we performed candidate *sod2*-targeting miRNAs predictions, reporter gene identification of putative complimentary region, gain-of-function and loss-of-function of miRNAs on SOD2 protein under H_2_O_2_ treatment, and identified a novel functional miRNA regulating SOD2 expression via repressing SOD2 protein translation.

## Materials and Methods

### Cell Culture

Rat PC12 cells and human SH-SY5Y cells (Shanghai Cell Bank, China) were separately cultured in high-glucose Dulbecco’s modified Eagle’s medium (DMEM) (Sigma, USA), supplemented with 10% calf serum (HyClone, USA), 100 U/ml of penicillin and 100mg/ml of streptomycin, and maintained in a humidified atmosphere of 5% CO_2_ at 37°C. Cells were subcultured every 2–3 days.

### H_2_O_2_ Treatment

The PC12 was seeded to a density of 1×10^5^/ml in 96-well plates, and 24 h later, the cell was treated with 12.5 µM, 25 µM, 50 µM, 100 µM and 200 µM H_2_O_2_ for 6 h, separately.

### Viability Assay

Viability of PC12 cells was measured by using MTT (3-(4, 5-dimethylthiazol-2-yl) 2, 5-diphenyl tetrazolium bromide) assay. Following incubation, the cells was aspirated and washed with the medium, and treated with 0.5 mg/L MTT (Sigma, USA). After 4 h incubation at 37°C, 100 µl DMSO (dimethyl sulfoxide) (Sigma, USA) was added to each well, then the 96-well plates were being shaked for 10 minutes and the absorbance at 590 nm of solubilized MTT formazan products was measured using a microplate reader (BioTek, USA).

### Real-time Polymerase Chain Reaction

Total RNA was extracted by TRIzol (Invitrogen, USA) according to the standard procedure. The RNA was treated with RNase-free DNase I (Takara, Japan), and subsequently reverse-transcribed with oligo dT and random 6mer primer. The expression of *sod2* mRNA relative to *β-actin* mRNA was determined by SYBR green I real-time quantitative PCR assay. The primer sequences were as follow, *sod2* forward: 5′-GCCTCCCTGACCTGCCTTAC-3′ reverse: 5′-GTGATTGATATGGCCCCCG-3′; *β-actin* forward: 5′-CCCTAAGGCCAACCGTGAA-3′, reverse: 5′-CCAGAGGCATACAGGGACAAC-3. The relative expression of miR-146a to 5sRNA was determined as described previously by Chen [23]. The primers were as follow, reverse transcription primer GTCGTATCCAGTGCAGGGTCCGAGGTATTCGCACTGGATACGACAACCCA; miR-146a forward primer: 5′-GCGTGAGAACTGAATTCCA-3′, reverse primer: 5′-GTGCAGGGTCCGAGGT-3′; 5sRNA forward primer: 5′-CAGCCATACCACCCTGAACG-3′, reverse primer: 5′-GGTATCCCAGGTGGTCTCCC-3′.

### Recombinant Plasmid Construction and Dual Luciferase Reporter Assay

The entire *sod2* mRNA 3′-UTR was PCR-amplified utilizing the sense (5′-GCTCTAGATCCGCCAGGCTGTGTGTC-3′) and antisense (5′-GCTCTAGAAGTAATGTGCATGCCTGGGG-3′) primers according to standard procedures and cloned into the *XbaI* site of the pGL3-Promoter plasmid (Promega, USA). Plasmid DNA was subsequently isolated from recombinant colonies and sequenced to ensure the authenticity and direction of the inserted *sod2* 3′UTR. For the luciferase reporter assays, PC12 cells were grown in DMEM with 10% FBS to 70% confluence in 48-well plates. Cells were transfected with 100 ng of firefly luciferase reporter vector containing the *sod2* 3′UTR (named pGL3-sod2-3′UTR) and miR-146a mimics (Genepharma, China) (final concentration was 100 nM) or negative control using lipofectamine 2000 (Invitrogen, USA). 10 ng pRL-TK vector (Promega, USA) was co-transfected as internal control for normalization of the transfection efficiency. After 24 hours, transfected cells were harvested with ice-cold phosphate-buffered saline, and dual luciferase assay was performed according to the manufacturer’s protocol (Promega, USA).

### MiRNA Transfection

miR-146a (rno-miR-146a and hsa-miR-146a share the same sequence) was synthesized by GenePharma company (China). For functional analysis, miR-146a (miR-146a) mimics, antisense miR-146a mimics, non-targeting miRNA mimics (negative control), and scrambled antisense miR-146a (antisense miRNA negative control) were transfected into PC12 and SH-SY5Y cells with lipofectamine according to the manufacturer’s instruction, separately. The final concentration of miR-146a mimics and non-targeting miRNA mimics was 100 nM, while the concentration of antisense miR-146a and antisense miRNA negative control was 33 nM. Cells were incubated for 48 h until lysis.

### Western Blot

After washing with cold PBS 3 times, cells were lysed in RIPA buffer of 150 mM NaCl, 10mM Tris-HCl, pH 7.4, 0.5% Triton X-100, and protease inhibitors (Sigma, USA), homogenized on ice, and centrifuged at 12,000 g at 4°C for 15 min. The supernatant was collected and stored at −80°C until use. Protein concentration was determined using the BCA Protein Assay Kit (Pierce, USA). 25 µg of protein extraction was loaded on 12% tris-polyacrylamide gels. The proteins were then transferred to nitrocellulose membrane (Milipore, USA). The membranes were blocked in 5% non-fat dry milk, washed in TBS with 0.05% Tween 20 (TBST), and incubated with mouse anti-SOD2 (Santa cruze, USA) in room temperature overnight at 4°C. Mouse anti-β-actin (Santa cruze, USA) was used for internal control. The membranes were washed three times in TBST and incubated for 1 hour with secondary antibodies conjugated to horseradish peroxidase, washed three times in TBST, and treated with Immun-Star HRP peroxide buffer and Luminol/Enhancer (Zhongshan, China) for chemiluminescence detection of protein bands.

### Statistical Analysis

Statistical analysis was done with one way ANOVA. Differences with *P*<0.05 were considered statistically significant.

## Results

### H_2_O_2_ Decreased the Survival Rate of PC12 Cells

**Figure 1:**
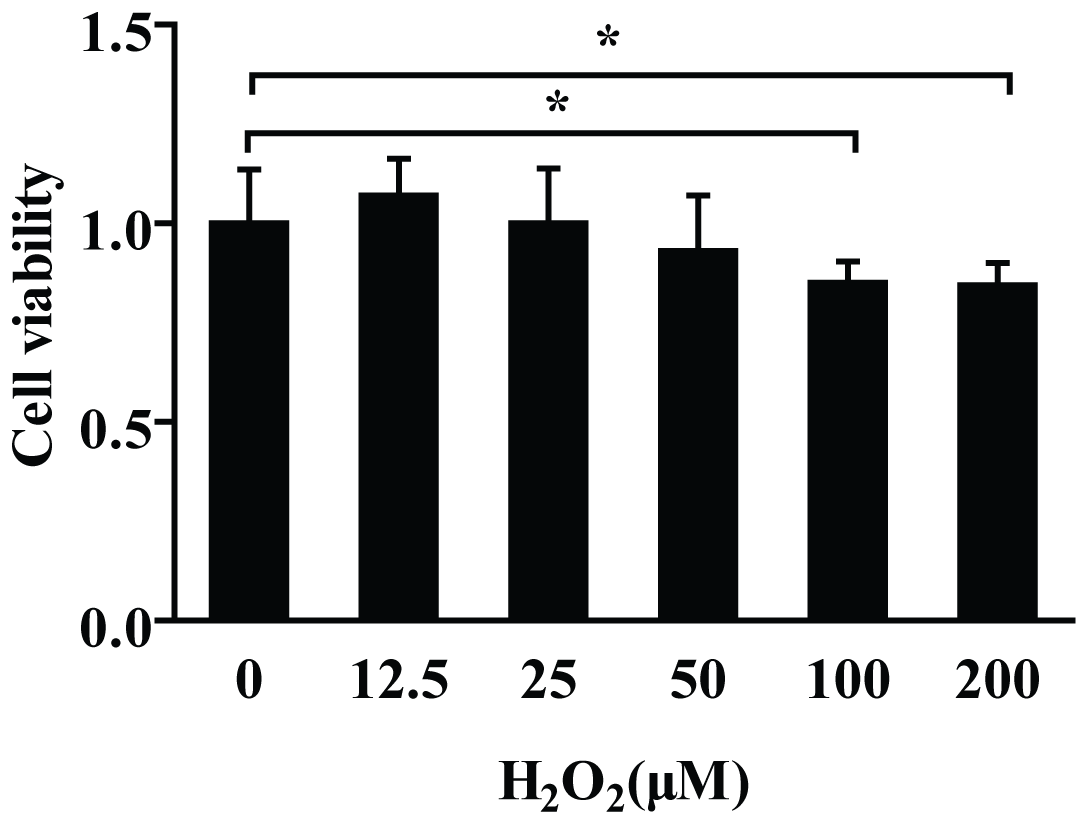
H_2_O_2_ decreased PC12 cells viability. MTT assay showed that 12.5∼25 µM H_2_O_2_ treatment had no obvious effect, while 50 µM, 100 µM, and 200 µM H_2_O_2_ treatment resulted in cell viability decrease by 7%, 15% and 16%, respectively. When compared with control group (0 µM), 100 µM and 200 µM group showed significant difference. Data are shown mean ± SD (n = 6). *P<0.05, as compared with control.

To investigate the survival rate changes of PC12 cells under the stimulation of H_2_O_2_, we treated the PC12 cells with different concentrations of H_2_O_2_ for 6 h. The results indicated that low concentrations of H_2_O_2_ (12.5 µM-25 µM) have no obvious effect on PC12 viability. While, with the increase of H_2_O_2_ concentrations, the viabilities of PC12 cells were gradually decreased significantly. (Fig. 1) Significant viability decrease was observed at 100 µM and 200 µM.

### H_2_O_2_ Regulated the Expression of SOD2 Protein

**Figure 2:**
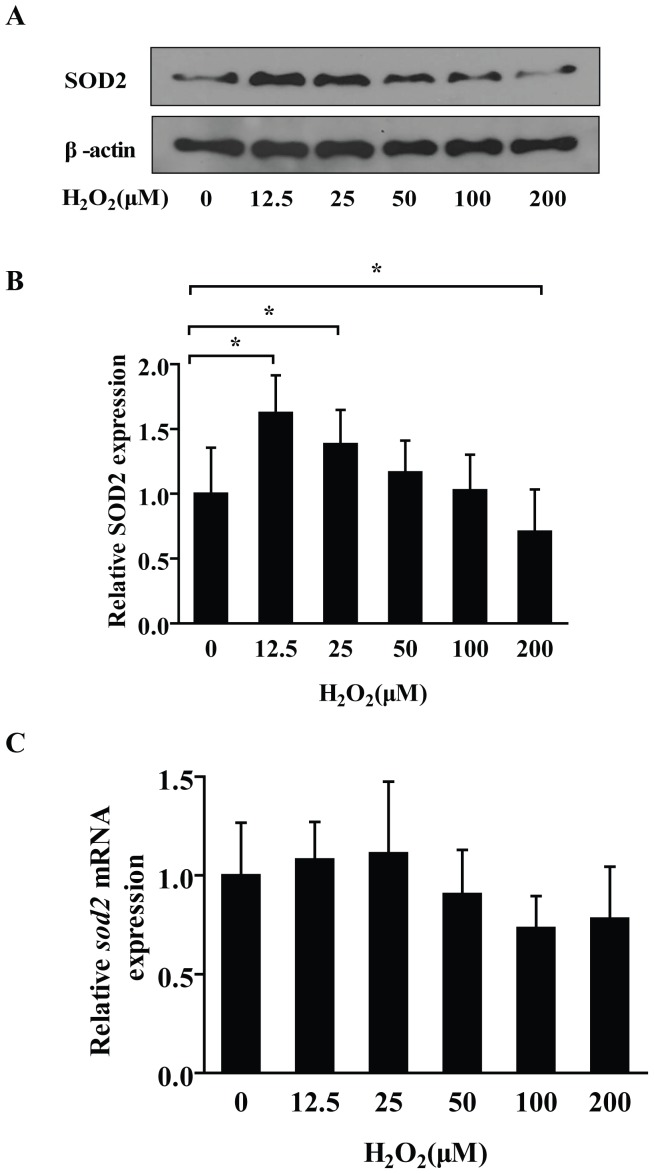
H_2_O_2_ down-regulated the expression level of SOD2 protein in a dose-dependent manner. (A) Along with the increase of H_2_O_2_ concentrations, the SOD2 protein was decreased gradually. Interestingly, the SOD2 protein showed an evident higher level with 12.5 µM and 25 µM H_2_O_2_ stimulation. With the higher concentration 200 µM H_2_O_2_ stimulation, it decreased significantly with a lower level than control group. (B) The relative amounts of SOD2, which were represented by the intensity ratio between SOD2 and β-actin in each lane. Data are shown mean ± SD from 3 independent experiments, each with 6 replicates. (C) H_2_O_2_ had no obvious effect on *sod2* mRNA expression in PC12 cells. After 6 h treatment with different concentrations of H_2_O_2_, the RT-PCR assay demonstrated there were no statistical differences of PC12 *sod2* mRNA expression levels between the groups. Data are shown the mean ± SD (n = 6). ANOVA (Dunnett) *P<0.05, as compared with control.

Our results demonstrated that, after 6 h treatment, low concentration (12.5 µM) of H_2_O_2_ could result in a relative high level of SOD2 protein. Evidently, the SOD2 protein levels were decreased gradually along with the elevation of H_2_O_2_ concentrations, and the SOD2 level in 200 µM was lower than the control group (Fig. 2A and 2B). Interestingly, there were no differences of *sod2* mRNA levels between each of the H_2_O_2_-treated groups (Fig. 2C).

### MiR-146a Interacted with the 3′UTR of *sod2* mRNA

**Figure 3:**
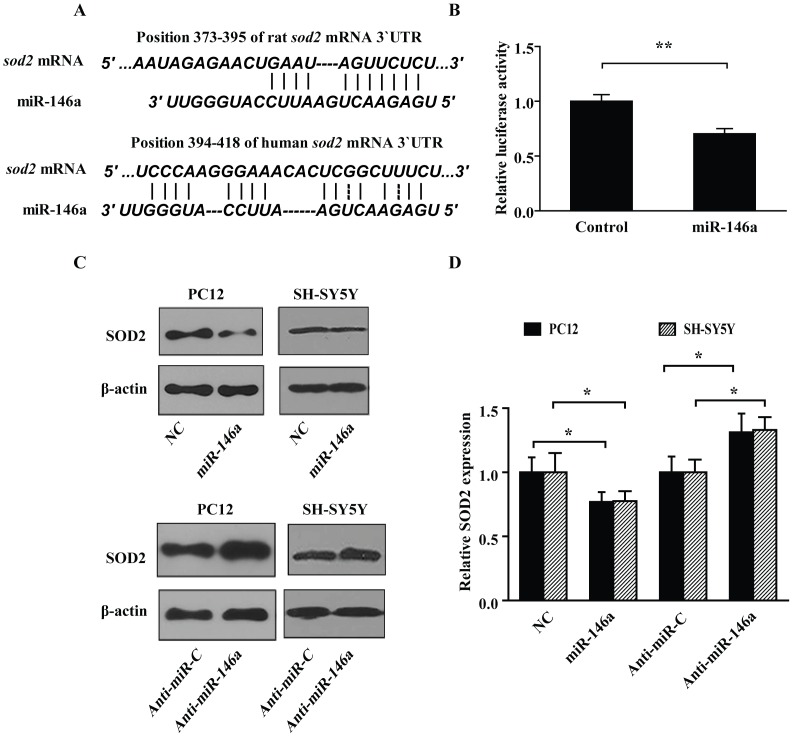
*SOD2* was identified as a target of miR-146a in PC12 and SH-SY5Y cells. (A) Prediction of the interaction between *sod2* mRNA 3′UTR and miR-146a. Sequence analysis showed that rat *sod2* mRNA 3′UTR 388-394 position mated with the rat miR-146a 2-8th nucleotides. (B) miR-146a down-regulated the luciferase activity of pGL3-Promoter-sod2-3′UTR. Dual luciferase reporter assay demonstrated that miR-146a mimics could down-regulate relative luciferase activity of pGL-sod2-3′UTR for 29.7%, compared with control group. Data are shown the mean ± SD (n = 6). **P<0.01, as compared with control. (C) miR-146a down-regulated the expression of SOD2 protein, while antisense-miR-146a reversely increased the amount of SOD2 protein. NC, non-targeting scrambled-miRNA control. Anti-miR-C, scrambled antisense miRNA control. Anti-miR-146a, antisense-miR-146a. (D) The relative amounts of SOD2 protein treated with miR-146a and antisense-miR-146a were presented by the intensity ratio between SOD2 and β*-*actin in each lane. Data are shown the mean ± SD from 3 independent experiments, *P<0.05.

With bioinformatics sequence analysis, we found a binding site of miR-146a seed region both in Rattus norvegicus and Homo sapiens *sod2* mRNA 3′UTR (Fig. 3A). To investigate whether miR-146a interacts with rat *sod2* mRNA 3′UTR *in vitro*, we inserted the whole length region of rat *sod2* mRNA 3′UTR into pGL3-promoter luciferase reporter plasmid 3′UTR region. The reporter constructs were co-transfected with miR-146a mimics in PC12 cells, and the results demonstrated that miR-146a mimics could down regulate the luciferase activity by 29.7% versus negative control group (Fig. 3B).

### MiR-146a Decreased SOD2 Expression

To investigate whether miR-146a participates in the regulation of SOD2 expression, we transfected miR-146a mimics or non-targeting miRNA mimics into PC12 and SH-SY5Y cells for 48 h, separately. Meanwhile, the antisense miR-146a mimics were transfected for the blockage of the internally originated miR-146a. The western blot assay showed that miR-146a mimics decreased the SOD2 protein expression significantly, while antisense miR-146a mimics up-regulated the SOD2 protein slightly, when compared with non-targeting scrambled-miRNAs and scrambled antisense miR-146a groups, respectively. Taken together, these results showed that miR-146a could regulate the SOD2 expression, and *sod2* was a target gene of miR-146a. (Fig. 3C and 3D). Also, these results in Homo sapiens cells as well as in Rattus norvegicus cells revealed the novel possible anti-oxidative role of miR-146a.

### H_2_O_2_ Induced miR-146a Expression in PC12 Cells

**Figure 4:**
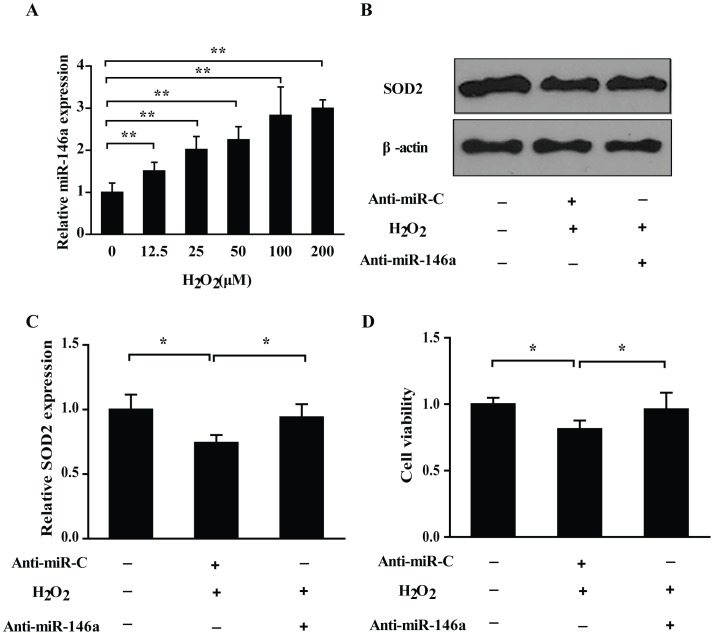
Identification of the regulatory role of miR-146a in the H_2_O_2_ induced changes of SOD2 protein and PC12 cells viability. (A) H_2_O_2_ increased the miR-146a expression in PC12 cells. Real-time RT-PCR revealed that H_2_O_2_ enhanced the miR-146a relative expression level in a dose-dependent manner in PC12 cells after the stimulation with H_2_O_2_ for 6 h. Data are shown the mean ± SD (n = 6), **P<0.01, as compared with control. (B) Down-regulation of SOD2 was reversed by antisense-miR-146a. PC12 cells were stimulated with 200 µM H_2_O_2_ after a pretreatment with antisense-miR-146a for 24 h. Western blot assay showed antisense-miR-146a reversed the SOD2 protein decrease induced by H_2_O_2_. (C) The relative amounts of SOD2 protein are presented by the intensity ratio between SOD2 and β-actin in each lane. Data are shown the mean ± SD from 3 independent experiments, *P<0.05. (D) Antisense-miR-146a retrieved the decrease of PC12 cells viability induced by 200 µM H_2_O_2_. Data are shown the mean ± SD (n = 6), *P<0.05.

As shown in Fig. 4A, miR-146a was up-regulated in a dose-dependent manner by H_2_O_2_ in PC12, which was inversely correlated with the SOD2 expression under H_2_O_2_ treatment. These results indicated the involvement of miR-146a in SOD2 protein expression regulation with H_2_O_2_ stimulation.

### Antisense-miR-146a Reversed the Decreased SOD2 Expression and PC12 Cells Viability

To verify whether the decrease of miR-146a possesses an effect on SOD2 expression and cell viability with H_2_O_2_ treatment, we pretreated PC12 cells with antisense-miR-146a mimics for 24 h, and then stimulated with 200 µM H_2_O_2_ for 6 h. The results demonstrated that antisense-miR-146a mimics pretreatment could reverse the down-regulated SOD2 expression (Fig. 4B and 4C) and decreased cell viability (Fig. 4D) by H_2_O_2_ in PC12 cells.

## Discussion

Our results indicate that high concentration of H_2_O_2_ causes cell viability decrease in PC12 cells, which is in accord with the previous study by Wei [24]. SOD2 plays an important role in maintenance of cell survival under stress conditions, and a lot of studies confirmed that cell viability decrease was correlated with SOD2 down-regulation under stress conditions [25], [26]. Meanwhile, when introduced exogenous SOD2 into cell the viability decrease could be improved more or less [27], [28]. To date, the efforts to decipher the mechanism of *Sod*2 gene expression are mainly focused on transcription and post-translational modification. Nevertheless, the post-transcriptional regulatory processes of *Sod*2 expression were seldom explored. Importantly, this regulatory process has been increasingly identified significant and necessary in the process of gene regulation, such as miRNAs down-regulation mechanism. Our studies demonstrate that H_2_O_2_ could significantly alter the SOD2 protein expression. Intriguingly, there are no differences of *sod2* mRNA levels between H_2_O_2_-treated group and control group. These results indicate that the variation of SOD2 protein under H_2_O_2_ treatment is mainly caused in SOD2 protein repression process other than mRNA degradation.

MiRNAs are a class of non-coding RNA, and recent years it has been proved that they play a pivotal role in gene expression regulation. MiR-146a is a widely expressed miRNAs in animal, and recently it has been confirmed to be involved in immunity, inflammation, viral infections, etc. [20]. With the bioinformatics tools, including RegRNA and Miranda, Dharap found that SOD2 was possibly a target of miR-145. After transient middle cerebral artery occlusion (MCAO), the rat post-ischemic brain showed highly expressed miR-145. Meanwhile, miR-145 inhibitor pretreatment could induce SOD2 expression in reperfusion brain, indicating that elevated miR-145 could depress the SOD2 expression in MCAO brain [29]. In addition, a study using luciferase reporter assay revealed that miR-222 could bind the *sod*2 3′UTR, and the expression of SOD2 was reduced in a carcinoma cells when transfected with ectopic miR-222 [30]. Also, Kriegel and colleagues demonstrated that treatment of human renal epithelial cells with TGF-β1 resulted in up-regulation of miR-382 and down-regulation of SOD2. In this study, knockdown of miR-382 could attenuate TGF-β1 induced down-regulation of SOD2 protein, and 3′UTR reporter assay showed miR-382 could target *sod*2 mRNA [31]. Taken together, these results suggest that miR-382, miR-145 and miR-222 can regulate the expression of SOD2 in different pathological processes.

In our study, the results of reporter assay indicate the interaction between miR-146a and *sod*2 3′UTR, and both the gain-of-function and loss-of-function experiments demonstrate that miR-146a could regulate the expression of SOD2. Furthermore, we confirm that miR-146a is up-regulated by H_2_O_2_, and H_2_O_2_-caused cell viability decrease can be reversed by antisense-miR-146a. These results identify the post-transcriptional regulatory role of miR-146a in SOD2 expression, indicating the possible involvement of miR-146a in oxidative stress.

In conclusion, this study demonstrates that *Sod2* expression can be regulated by miR-146a, and identifies a novel function of miR-146a in *sod*2 expression via inhibiting protein translation.
